# Antiproliferative effects of AAV-delivered CRISPR/Cas9-based degradation of the HPV18-E6 gene in HeLa cells

**DOI:** 10.1038/s41598-022-06025-w

**Published:** 2022-02-09

**Authors:** Zahra Noroozi, Mehdi Shamsara, Elahe Valipour, Sahar Esfandyari, Alireza Ehghaghi, Amir Monfaredan, Zahra Azizi, Elahe Motevaseli, Mohammad Hossein Modarressi

**Affiliations:** 1 Department of Molecular Medicine, School of Advanced Technologies in Medicine, Tehran University of Medical Sciences, Tehran, Iran; 2 Department of Animal Biotechnology, National Institute of Genetic Engineering and Biotechnology, Tehran, Iran; 3 Department of Medical Genetics, Faculty of Medicine, Tehran University of Medical Sciences, Tehran, Iran; 4 Department of Urology, College of Medicine, University of Illinois at Chicago, Chicago, IL USA

**Keywords:** Biochemistry, Biological techniques, Cancer, Cell biology, Genetics, Molecular biology, Diseases, Molecular medicine

## Abstract

Human papillomavirus infections are associated with most cervical cancers, which are the fourth most common cancer in women. HPV-E6 protein binds to protein p53 and inhibits its function, leading to the switching of normal cells toward cancer cells. Here, we disrupted the HPV-E6 gene and investigated its effects on the proliferation and apoptosis of HeLa cells. The HPV18-E6 gene was targeted with two designed sgRNAs cloned into an AAV-CRISPR-based plasmid. The AAV-E6-CRISPR/Cas9 virions were prepared and titrated in HEK293t cells. The cleavage created in the HPV-E6 gene was detected using the T7E1 assay. Cell cycle profiling, MTT assay, and annexin V/PI staining were performed. Also, the p53 protein level was measured by Western blotting. Our data showed that disruption of the HPV-E6 gene led to increased cell apoptosis and decreased cell proliferation. A significant accumulation of infected cells in sub-G1 phase was observed in the cell profiling assay. Also, HPV-E6 gene disruption resulted in a significant increase in the level of P53 protein. Our findings indicated that AAV-mediated delivery of CRISPR/Cas9 can effectively target the HPV-E6 gene in HeLa cells, and its antiproliferative effects may provide therapeutic benefits of local administration of this gene-editing system for HPV-related cervical cancers.

## Introduction

Cervical cancer is the fourth most common cancer in women and the fourth leading cause of cancer death, with an estimated 604,000 new cases and 342,000 deaths in 2020^1^**. **Almost all cases of cervical cancer (99%) are associated with infection with high-risk human papillomaviruses (HPVs)^2^. HPVs are a group of carcinogenic DNA viruses with over 200 different kinds, although not all of them are associated with health problems^3^. Most HPV-related health problems are usually caused by types 6, 11, 16 or 18, which in particular type 16 and 18 are involved in the formation of cervical tumors and precancerous lesions of the cervix^4^.

Oncogenic proteins E6 and E7 are considered to be the main causes of HPV-related cervical carcinoma^5^, which are expressed immediately after the fusion of viral episodic DNA into cellular genomic DNA^2^. One of the main functions of these proteins is to inhibit tumor suppressor proteins such as p53 and Rb, which cause host cells to switch toward cancerous cells^6,7^. They also support the uncontrolled proliferation of infected cells and the development of cancer. Accordingly, the inactivation of viral oncogenes E6 and E7 is a promising therapeutic target for HPV-positive cancers.

The clustered regularly interspaced short palindromic repeats (CRISPR)/CRISPR-associated 9 (Cas9) system is a new evolving technology that has made it possible to modify genes in a variety of species^8^. This powerful genome-editing tool consists of single guide RNAs (sgRNAs) and the effector protein Cas9^9,10^. Under the guidance of sgRNAs, Cas9 endonuclease creates double-strand breaks (DSBs) at a specific site of targeted DNA complementary to guide RNA. These DSBs are then repaired by the error-prone nonhomologous end joining (NHEJ) repair pathway, leading to insertions or deletions (indels) or frameshift mutations in the targeted gene^11–13^.

CRISPR/Cas9 system can enter cells in the form of DNA, mRNA, or protein. DNA encoding gRNA and Cas9 can be delivered into cells using plasmids or viral vectors, like adeno-associated viruses (AAVs)^14,15^. AAVs are an excellent gene transfer tool with a long history of success in clinical trials^16^. AAV-mediated delivery of CRISPR/ Cas9 has been used for various genetic disorders that have been associated with satisfactory consequences of gene editing^13,15–17^.

In this research, we applied the AAV-E6-CRISPR/Cas9 system to disrupt the gene encoding oncogene E6 in the HPV18-positive HeLa cell line. We demonstrated that the cleavage of HPV18-E6 oncogene led to increased cell apoptosis and decreased cell proliferation rates. Disruption of the E6 gene and thereby loss of oncoprotein E6 restored p53 expression. Our data showed that the HPV18-E6-guided AAV-CRISPR/Cas could work as a potential therapeutic strategy for cervical cancer.

## Results

### Editing tool preparation

**Figure 1:**
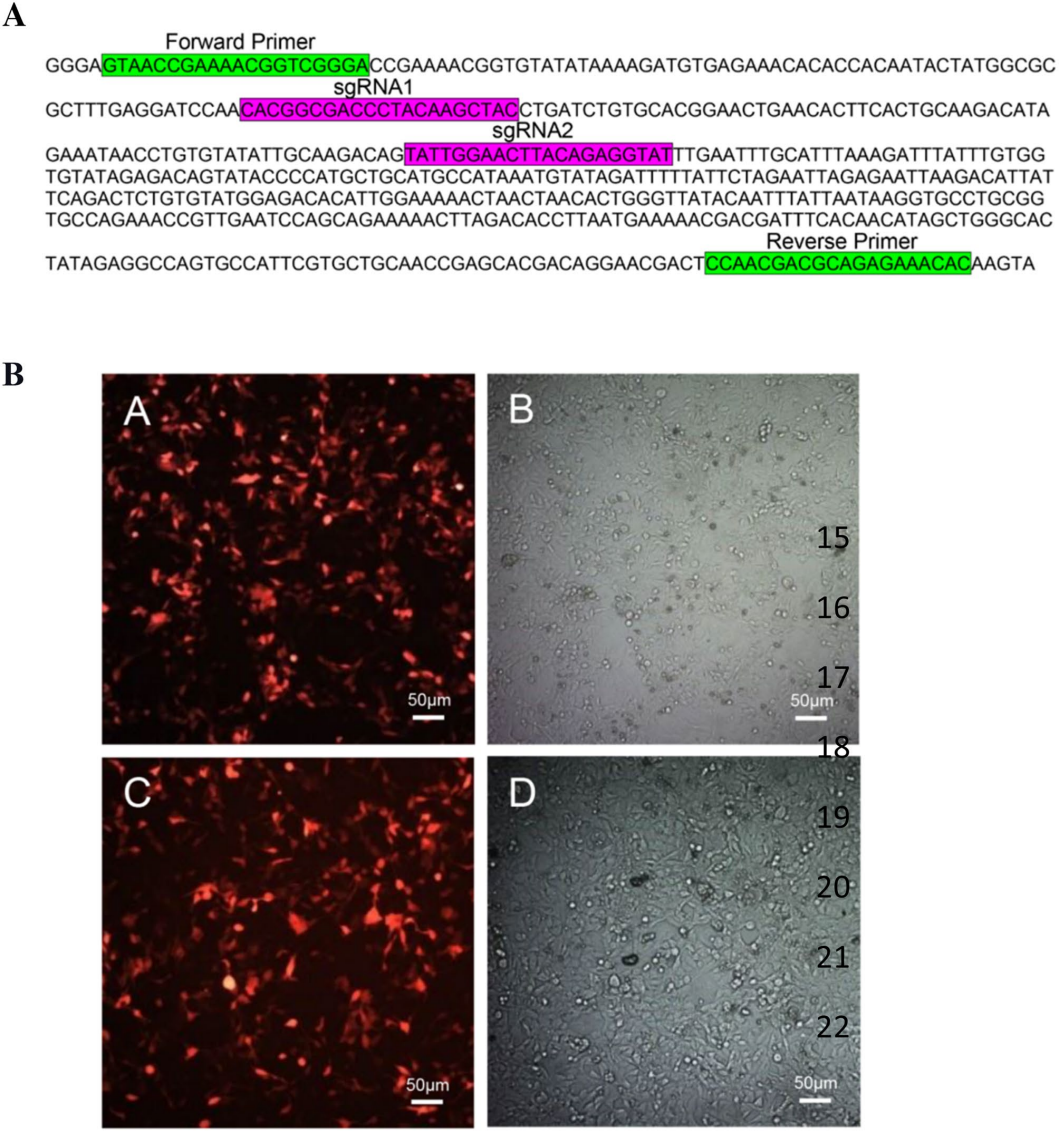
Editing tool preparation. (**A**) The position of two sgRNAs and the primers used in this study. sgRNA-1 for minus strand, and sgRNA-2 for plus strand of the gene. (**B**) To optimized cell transduction, RFP expression was visualized directly using fluorescence microscopy (Scale bars = 50 μM). (**A**) HeLa cells infected with AAV-E6 sgRNAs/Cas9 virions and (**B**) Bright field. (**C**) HEK293t cells co-transfected with triple vectors PX601-mCherry, pHelper, and pAAV-RC for production of AAV-E6-CRISPR/Cas9 particles and (**D**) Bright field.

In order to investigate the effect of the AAV-mediated delivery of CRISPR/Cas9 editing tool on the viral E6 gene, two sgRNAs were designed and cloned into the pX601 AAV transfer vector encoding SaCas9 protein fused with mCherry. The position of two sgRNAs and the test primers used in this study are shown in Fig. 1A.

AAV particles were generated in HEK293t cells and concentrated through the amicon filters (Millipore). The quantification of virus titers was performed using qPCR as 5.52 × 10^8^ virus genome per milliliter. The HeLa cells were transduced with a multiplicity of infection (MOI) of about 30. The transduction efficiency was determined by fluorescence imaging followed by the ImageJ analysis. As shown in Fig. 1B, the cells with red fluorescence emission were accounted for approximately 80% and 90% for HeLa and HEK293t cells, respectively.

### Molecular evidence of the gene editing using AAV-E6-CRISPR/Cas9

**Figure 2:**
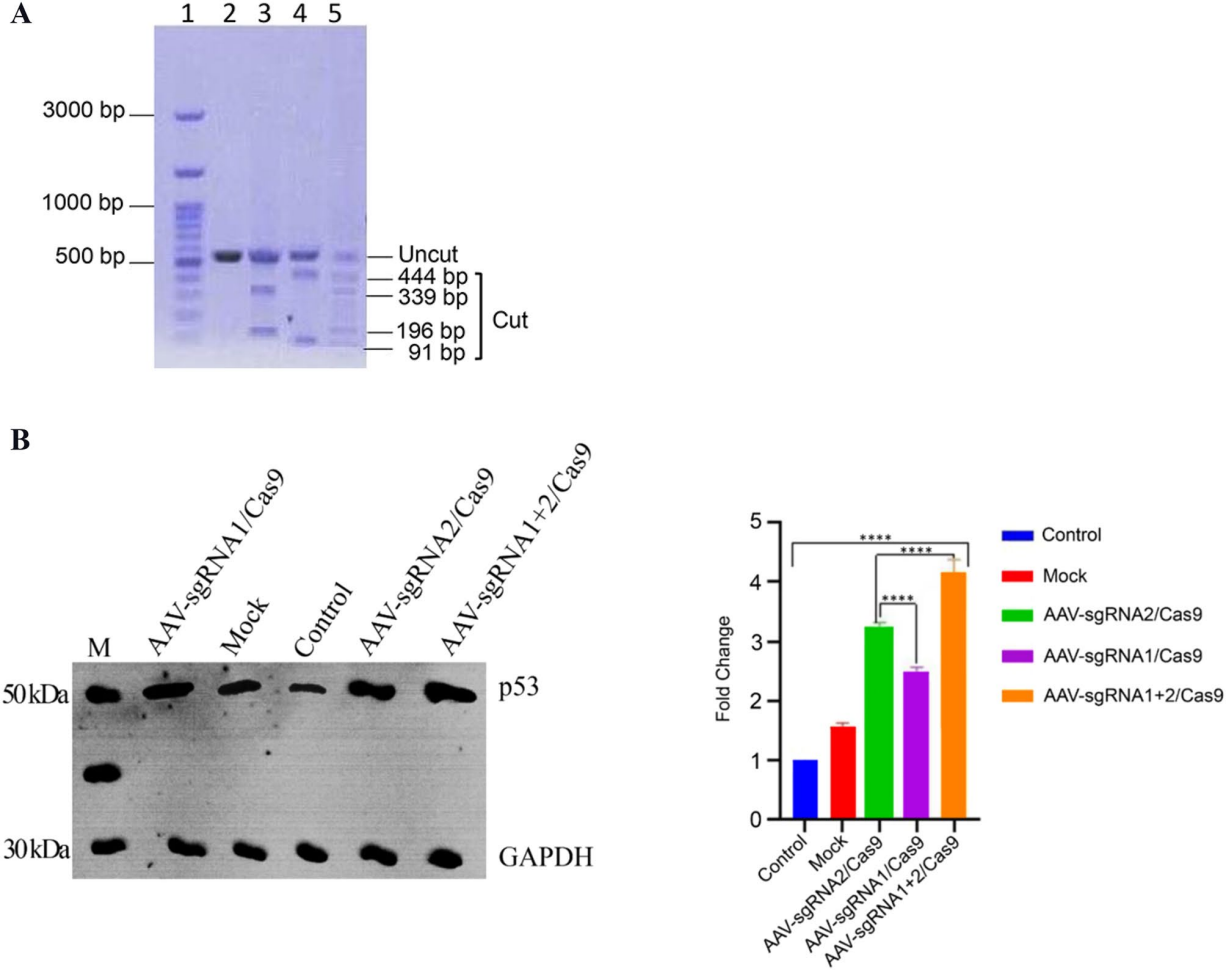
Molecular evidence of the gene editing. (**A**) The function of AAV-E6-CRISPR/Cas9 in HeLa cells was assessed using the endonuclease activity of T7E1. Lane 1: 100 bp DNA molecular weight marker; lane 2: untreated cells (control); Lanes 3–5 show the results of T7EI treatment. As the result of T7EI treatment, heteroduplex DNAs showed two cleaved products at 339 bp and 196 bp for the cells infected with AAV-E6-sgRNA-2/Cas9 (lane 3), two bands at 444 bp and 91 bp for the cell infected with AAV-E6-sgRNA-1/Cas9 (lane 4) and four bands of 91 bp, 196 bp, 339 bp, and 444 bp for the cells infected with AAV-E6-sgRNA1 + 2/Cas9 (lane 5). (**B**) The p53 protein level in HeLa cells was measured using western blotting on day 5 after transduction. GAPDH was used as an internal control. The ImageJ software was used to measure the p53:GAPDH ratios in HeLa cells. The expression level of p53 protein was significantly increased in cells infected with AAV-E6-CRISPR/Cas9 compared with the control and mock cell groups (****p < 0.0001). The expression level of GAPDH was used to normalize the band intensity (Supplementary S1).

The T7E1 assay was applied to evaluate the AAV-E6-CRISPR genome editing activities. This enzyme detects and cleaves mutations at mismatch sites. The flanking region of sgRNA-1 and 2 recognition site in the length of 531 bp was amplified using a set of specific primers during PCR (Fig. 2A). T7E1-treated heteroduplexes were separated by electrophoresis and the agarose gels were imaged and analyzed by ImageJ software for determining the intensity of cleaved bands to intact ones. The cleavage efficiency with sgRNA-1 and sgRNA-2 and mix of two sgRNA (sgRNA1 + 2) was measured 32%, 39%, and 56%, respectively.

Also, HPV-E6 mediates the degradation of tumor suppressor protein p53 in host cells. Therefore, Western blotting was performed to investigate whether inactivation of the E6 gene could affect the p53 protein level. Western blotting results (Fig. 2B, and supplementary S1 data) showed that gene editing using AAV-E6-CRISPR/Cas9 resulted in increased p53 protein levels. The p53 protein levels were significantly upregulated in cells transduced with AAV-E6 CRISPR/Cas9 compared to the control and mock cell groups (*P* < 0.0001). Increased levels of p53 protein were 4.1-, 3.2- and 2.4-fold in cells receiving sgRNA-1, sgRNA-2, and both sgRNAs, respectively, compared to the mock and control cell groups. No significant difference was observed in p53 protein levels between the control and mock cell group.

### Gene editing using AAV-E6-CRISPR/Cas9 increased cell apoptosis

**Figure 3:**
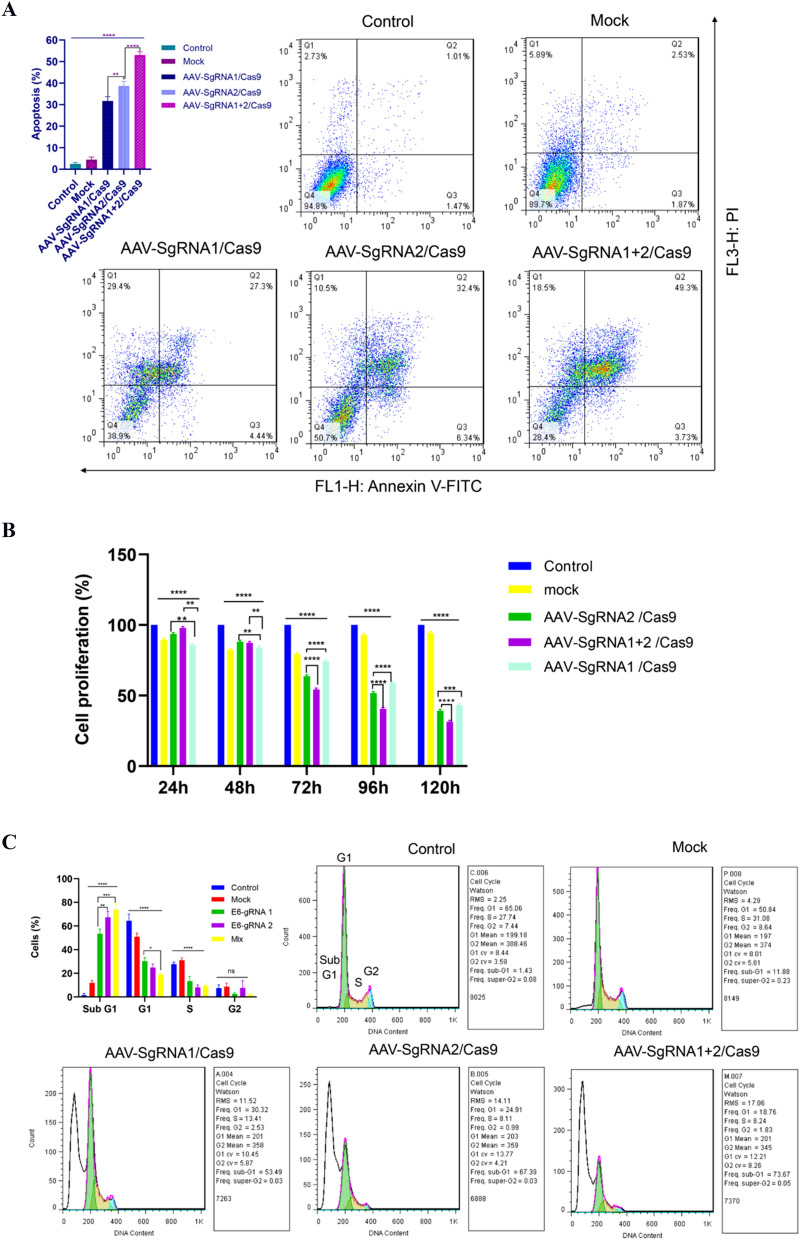
The effect of the gene editing on cell apoptosis (**A**) and proliferation (**B**,**C**). (**A**) Cells were double-stained with propidium iodide and annexin V-FITC, then the rate of apoptosis was monitored by flow cytometry after 5 days after infection of HeLa cells. Flow cytometry finding indicated that AAV-E6-CRISPR/Cas9 infection significantly induced apoptosis in HeLa cells compared to the control and mock cell groups. (**B**) MTT assay showed that disruption of the E6 gene using AAV-E6-CRISPR/Cas9 could significantly inhibit cell proliferation in HeLa cells. (**C**) Cell cycle profiling showed that AAV-E6-CRISPR/Cas9-mediated inactivation of the E6 gene resulted in cell cycle arrest in sub G1. The represented data are as mean ± SD independent experiments. Data were analyzed using a one-way ANOVA test. *p* < 0.05 was used to determine statistical significance (**p-value < 0.01 ***p-value < 0.001 ****p-value < 0.0001).

The effect of CRISPR-mediated editing of the HPV-E6 gene on cell apoptosis in HeLa cells was studied. Flow cytometry findings revealed a significant increase in the rate of cell apoptosis due to the delivery of E6-specific CRISPR components. As shown in Fig. 3A, 27.3% of the cells infected with sgRNA-1 and 32.4% of the cells infected with sgRNA-2 were in the early apoptosis stage. Total apoptotic cells in these two groups were 31.74% and 38.74%, respectively. The percentage of apoptotic cells in the cells receiving both sgRNA-1 and 2 was 49.3% for early apoptosis and 53.03% for total apoptosis. No difference was observed between the control cell group and the mock cell group.

### Gene editing using AAV-E6-CRISPR/Cas9 decreased cell proliferation rate in a time-dependent way

The disruption effect of the E6 gene on the proliferation of HeLa cells was evaluated using the MTT (Fig. 3B) and cell cycle profiling (Fig. 3C). HeLa cell proliferation was measured at five points of time of transduction and compared with the mock and control cell groups. A significant time-dependent decrease was observed in the proliferation of the cell groups transduced with AAV-E6-CRISPR/Cas9 (Fig. 3B). Moreover, among the cell groups receiving AAV-E6 CRISPR/Cas9, the highest reduction in cell proliferation was observed in the cell group transduced with both sgRNA-1 and 2 as 31.42% at 120 h of transduction. At the same time, the percentage of cell proliferation in cells transduced with sgRNA-1 and sgRNA-2 was 43.3% and 39.15%, respectively.

To confirm the MTT results, cell cycle profiling was performed. Transduction of HeLa cells with AAV-E6-CRISPR/Cas9 resulted in cell accumulation in sub-G1 phase (Fig. 3C). By treating the cells with AAV expressing sgRNA-1 and sgRNA-2, 53.49% and 67.39% of the cells were accumulated in sub-G1 phase, respectively. This percentage was 73.67% for the cells receiving both sgRNAs, which was higher than the cells receiving each sgRNA alone. Whereas, the percentage of cells accumulated in sub-G1 phase was 11.88% and 1.43% respectively for the mock and control cell groups. Overall, the results of MTT assay and lower cell accumulation in phases S and G2 in cell cycle profiling in AAV-E6-CRISPR/Cas9-infected cells indicated that HPV-E6 gene disruption significantly reduced cell proliferation and led to cell cycle cessation in sub-G1 phase.

## Discussion

The aim of this study was to inactivate the gene encoding HPV-E6 protein in HeLa cells, which is a line derived from cervical cancer. HPV-E6 oncoprotein mediates the degradation of cell cycle controlling p53 protein by binding to it, thereby leading to uncontrolled cell growth and cell switching to cancer. Therefore, HPV-E6 plays a pivotal function in initiating and maintaining the malignant phenotype of cervical cancer cells^2,18,19^. Targeted inactivation of the E6 gene using gene-editing tools, such as CRISPR/Cas9, appears to be an inhibitory way against cervical cancer cell growth and even be able to restore the normal phenotype.

Today, genetic modification-mediated therapies have created a wide field of research for the treatment of viral infectious diseases and virus-related cancers^20^. Here, we designed a CRISPR/Cas9 AAV vector to combat HPV-related cancer cells. The extrachromosomal entity of AAV without genome integration property makes AAV more suitable than vectors based on integrative lenti- and retro-viruses for cancer genetic-based therapies^21^. Our results showed that loss-of-function of E6 protein in HeLa cells is a promising therapeutic strategy for defense against HPV-induced cancer phenotype. In order to inactivation of HPV-E6 oncoprotein, two sgRNAs with high specificity and high cleavage efficiency were designed and cloned separately into an AAV-based vector. In this study, we used the AAV2 serotype to transfer sgRNAs into HeLa cells. Previous studies have shown the efficiency of this serotype for gene transfer into cervical cancer cells^22,23^.

The cutting efficiency of the sgRNAs was evaluated by PCR and the T7E1 assay after transduction of HeLa cells with AAV-E6-CRISPR/Cas9. Our results showed that the designed sgRNAs could efficiently target and cleave the E6 gene in the cervical cancer HeLa cells.

E6 protein binds to and destroys p53 protein and thereby as a result, it prevents host cells from apoptosis. Disruption of the HPV-E6 gene using editing tools can restore the activities dependent on p53 protein and induce apoptosis in cancer cells. Activated p53 controls cell cycle progression by upregulating p21 protein, which in turn inhibits cyclin‐dependent kinase 2 and decreases G1/S transition^7^. Consistent with these findings, our results indicated that AAV-E6-CRISPR/Cas9 caused cell cycle arrest in sub-G1 phase and decreased cell proliferation. In addition, flow cytometry data revealed an increase in cell apoptosis rate due to AAV-E6-CRISPR/Cas9, which was consistent with the observed increased p53 protein level.

Our data were consistent with previous studies suggesting HPV-E6 oncogene as one of the factors involved in maintaining the malignant phenotype of cervical cancer cells. Ling et al.^7^ used a non-viral delivery system for CRISPR/Cas9 to target the HPV18-E6 and E7 genes and reported that E6E7- knockout induced cell apoptosis and significantly inhibited cell proliferation in vitro.

Kennedy et al.^2^ in a study reported that Lentivirus carrying CRISPR/Cas9 specific for the HPV18-E6 and E7 genes induce the death of cervical carcinoma cells. They suggested the development of AAV-based vectors for the successful eradication of HPV cancers in vivo. In a similar study, Wang et al.^5^ confirmed that a Lentivirus-CRISPR/Cas9 system targeting HPV18-E6 or E7 genes effectively blocked the expression of oncogenes E6 or E7 in HeLa cells, resulting in cell cycle arrest and decreased cell proliferation. Zheng et al.^17^ reported that HPV16 positive human cervical cancer cells transfected with E7-specific gRNA/Cas9 could disrupt the E7 gene at specific sites, inducing apoptosis and inhibiting the growth of HPV positive SiHa and Caski cells. Also, they showed downregulation of HPV16-E7 lead to upregulation of tumor suppressor protein pRb and inhibition of cell proliferation. Our observations were consistent with the results of these studies.

Various viral, non-viral (for example, lipid nanoparticles), and physical (for example, hydrodynamic injection) based delivery approaches of the CRISPR/Cas9 complex have been adopted for in vivo genome editing. But because AAV-based delivery systems offer major benefits, they have attracted a lot of attention, especially for therapeutic purposes. Also, AAV can exist long term as concatemers in non-dividing cells for stable transgene expressions.

In addition to the episomal nature of AAV rather than random insertion into the host genome and reducing the likelihood of adjacent gene dysfunction and mutagenicity, the low immune response and toxicity induced by AAV reported in various studies on animal models are among the cases that highlight the use of AAV-based delivery systems for in vivo genome editing. Indeed, there has been no reported case of disease caused by AAV in humans. So, good safety profile and high therapeutic efficacy of AAV in a wide range of animal models and human clinical trials have emphasized AAV-based viral vectors as one of the most suitable viral vectors for gene therapeutic applications and gene transfer in vivo.

On the other hand, one of the main limitations of systems AAV for simultaneous loading sgRNA and Cas9 is actually the size of the construct, which limits the use of large Cas. To overcome this limitation, we used PX601 mCherry vector for simultaneous transmission of sgRNA and Cas9 using a AAV-based vector. PX601 mCherry vector Carries a Cas9 belongs to a species of *Staphylococcus aureus* and smaller than the other Cas9.

Also, Yoshiba et al.^22^ used AAV-based CRISPR/Cas9 system for disruption of the E6 gene in HeLa, HCS-2, and SKG-1 cell lines. In fact, they first transfected these cell lines with a construct carrying the Cas9 gene and then selected cells that were stably transfected using colony selection under an antibiotic condition. So, they first created Cas9 nuclease-expressing cells and then transfected these cells with the AAV vector carrying sgRNA. It is clear that the process of creating stably transfected cell lines is time consuming. And importantly, the colony selection process for obtaining the desired cells is limited to in vitro and it is practically impossible to use this method in vivo. Whereas in our proposed method, Cas9 and sgRNA are both simultaneously transported into the cell using an AAV particle and there is no need to create a Cas9-expressing cells. The method used in our study, unlike the study of Yoshiba et al., is not time consuming and can be applied in vivo. On the other hand, Yoshiba et al. used only one sgRNA to prove their hypothesis, while we examined two sgRNAs independently as well as their combination simultaneously, which showed better effects than individual sgRNAs.

In conclusion, our results showed that the use of AAV for CRISPR/Cas9 delivery could successfully infect HeLa cells, specifically cleaved the HPV-E6 oncogene, reversed the malignant phenotype, and increased p53 protein level. Overall, delivery of the CRISPR/Cas9 system with AAV is capable of eliminating HPV infection and may be useful for local treatment of HPV-related cervical cancer.

## Methods

### sgRNA designing and cloning

A set of sgRNAs were designed and analyzed using the ZiFit Web application (http://zifit.partners.org/). The position of two sgRNAs and the test primers used in this study are shown in Fig. 1A. We investigated CRISPOR database for information about off-Targets of designed sgRNAs, and selected sgRNAs with no predicted off-target in exonic regions. Considering that they have the most efficient and the least off-target scores, two sgRNAs were selected to target the HPV18-E6 gene; sgRNA-1 with the sequence 5’- GTAGCTTGTAGGGTCGCCGTG-3’ for minus strand, and sgRNA-2 with the sequence 5’- TATTGGAACTTACAGAGGTAT-3’for plus strand of the gene. The sgRNAs were synthesized as oligonucleotide DNA sequences and cloned into a PX601 mCherry plasmid (Addgene, Cat no: 84039) expressing *Staphylococcus aureus* Cas9 protein (SaCas9). Conjugation of SaCas9 to mCherry marker makes it useful for fluorescence microscopy analysis. Also, this plasmid contains the AAV-2 inverted terminal repeats (ITRs) that regulate viral replication and packaging.

### Cell culture

Cell lines HeLa and HEK293t were purchased from the Pasteur Institute, Tehran, Iran. HeLa cells contain HPV18 DNA in their genome and express oncoproteins E6 and E7. HEK293t cell line, a human embryonic kidney cell line, was used for packaging and producing replicative-deficient AAV-E6-CRISPR/Cas9 virions through co-transfection of three AAV Helper-Free System plasmids; an ITR-containing plasmid, pAAV-RC, and pHelper. HeLa and HEK293t cells were cultured under high glucose Dulbecco’s modified Eagle medium (DMEM) containing 10% FBS and 1% Penicillin/Streptomycin (Sigma-Aldrich, USA) at 37 °C/5% CO_2_.

### Production of AAV-E6-CRISPR/Cas9 particles

We used the AAV2 serotype to transfer sgRNAs into HeLa cells. AAV2 particles were packaged using the triple transfection method. First, a transfection mixture consisting of 14 µg pHelper (Cell Biolabs), 8 µg pAAV-RC2 (Cell Biolabs), 8 µg PX601-mCherry, and 60 µg Polyethylenimine (PEI) (sigma, 25 kDa, 1 mg/ml in H_2_O, pH 7) was prepared in cell culture medium and incubated at room temperature for 15 min. Then, the mixture was drop-wised on 70–80% confluent HEK293t cells cultured in 10-cm plates. One day after transfection, the culture medium on the cells was replaced with a fresh medium (high glucose DMEM containing 2% FBS). Three days after transfection, the cells were harvested to release viral particles in three successive cycles of cooling on dry ice and heating in a 37° C water bath. Cell debris was collected by centrifugation at 10,000×*g* for 10 min at room temperature. AAV-containing lysates were treated with DNaseI for 30 min at 37 °C. The viral particles were then concentrated using 15 mL of 100 kDa molecular weight cut-off filters^24^. AAVs were titrated using the quantitative polymerase chain reaction (qPCR) by ITR forward primer 5'-GGAACCCCTAGTGATGGAGTT-3’ and reverse primer 5'-CGGCCTCAGTGAGCGA-3’, according to Aurnhammer et al. protocol^25^.

### T7 endonuclease I (T7E1) assay

The T7E1 assay was used to evaluate the cleavage of the E6 gene created by AAV-E6-CRISPR / Cas9. Briefly, 2 × 10^5^ cells/well of HeLa cells were seeded in 6-well plates and transduced by AAV-E6-CRISPR/Cas9 the next day. Here five cell groups were defined including control (untreated), mock (cells infected with an AAV–Cas9 without any sgRNA), cells infected with AAV-E6-sgRNA-1/Cas9, cells infected with AAV-E6-sgRNA-2/Cas9, and cells co-infected with AAV-E6-sgRNA-1/Cas9 and AAV-E6-sgRNA-2/Cas9 (AAV-E6-sgRNA-1 + 2/Cas9). The genomic DNA of the cells was extracted 72 h after infection using a phenol–chloroform method. The HPV18-E6 gene was amplified using PCR with the E6 forward 5’- GTAACCGAAAACGGTCGGGA-3’and reverse 5’- GTGTTTCTCTGCGTCGTTGG-3’ primers.

To form heteroduplex DNAs, the PCR products were denatured at 95 °C and hybridized by stepwise descending temperature. 200 ng of heteroduplexes were digested with two units of T7 endonuclease 1 (New England BioLabs) for 15 min at 37 °C and separated on 2% agarose gel. The cleavage efficiency of sgRNAs was calculated using the ImageJ and the following equation: gene modification (%) = 100 × [1 − (1 − fraction cleaved)1/2], where the fraction cleaved is the overall relative density of the cleavage bands divided by an accumulation of relative density of the cleavage bands and uncut bands^18^.

### MTT assay

4.0 × 10^3^ cells/well were seeded in a 96-well plate in triplicate and then transduced by the packaged AAV-E6-CRISPR/Cas9. The infected cells were incubated in the complete DMEM at 37 °C/5% CO2. Every day a set of wells were subjected for the MTT assay. 40 µl of 5 mg/ml 3-(4,5-dimethylthiazol-2-yl)-2,5-diphenyltetrazolium bromide solution (Sigma, USA) was added to each well and stored at 37 °C for 2.5 h. The precipitated formazan crystals were dissolved in 160 μL DMSO and the absorbance was measured using an ELISA reader at 570 nm.

### Annexin V/PI staining

Flow cytometry was performed for analysis of Annexin V-FITC / propidium iodide (PI) staining to evaluate cell apoptosis.5.0 × 10^5^ HeLa cells/well in 6-well plates were transduced with the AAV-E6-CRISPR/Cas9 virions. Five days of transduction, the cells were trypsinized and double-stained with annexin V/PI using PI Annexin V Apoptosis Detection Kit and BD FACS Calibur system (BD biosciences, San Jose, CA, USA) according to the instruction given by the manufacturer. The results were analyzed using the FlowJo software.

### Cell cycle profiling assay

Flow cytometry technique was used to obtain the cell distribution profile. 5.0 × 10^5^ cells/well in a 6-well plate were transduced with the AAV-E6-CRISPR/Cas9 virions. After 5 days of transduction, the cells were collected and stained with 1 ml of PI master mix (PBS 950 µl, RNase 10 µl, PI 40 µl) and incubated for 30 min at room temperature. Using a flow cytometer (BD biosciences, San Jose, CA, USA), the distribution of cell cycle was assessed and the data were analyzed using the FlowJo software**.**

### Western Blotting

Protein extraction from the AAV-E6-CRISPR/Cas9-transduced HeLa cells was performed using RIPA buffer. 5 μl of each cell lysate was run on 20% SDS-PAGE and then transferred to polyvinylidene difluoride (PVDF) membranes. The membranes were blocked with 5% skim milk in Tris-buffered saline–Tween 20 and then incubated with an anti-p53 primary antibody (Abcam, Cat no. ab131442) and anti-GAPDH (Abcam, Cat no. ab128915). The PVDF membranes were washed three times and incubated with a corresponding HRP-conjugated secondary antibody. The immunoreactive bands were visualized with a chemiluminescence detector and then imaged. The band intensity was quantified using the ImageJ software.

### Statistical analysis

The data were analyzed using Graph Pad Prism software 8.0.2. One-way ANOVA was used for the statistical analysis of various groups. Data were presented as means ± standard deviation. *p*-value of less than 0.05 was considered statistically significant.

## Supplementary Information

Supplementary Information.
