# Supplementary material for: Antiproliferative effects of AAV-delivered CRISPR/Cas9-based degradation of the HPV18-E6 gene in HeLa cells
Source: Sci Rep. 2022 Feb 9;12:2224. doi: 10.1038/s41598-022-06025-w (PMC8828776; doi:10.1038/s41598-022-06025-w)
Supplement: Supplementary file 1 — Supplementary Information. [file 41598_2022_6025_MOESM1_ESM.docx]

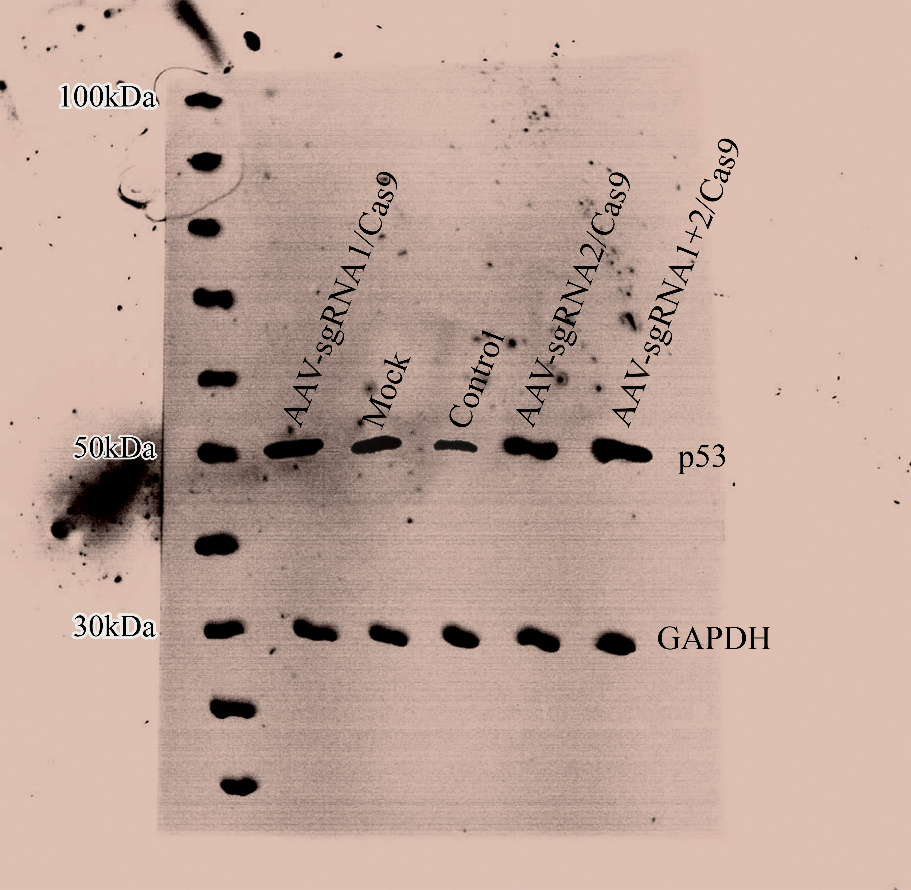


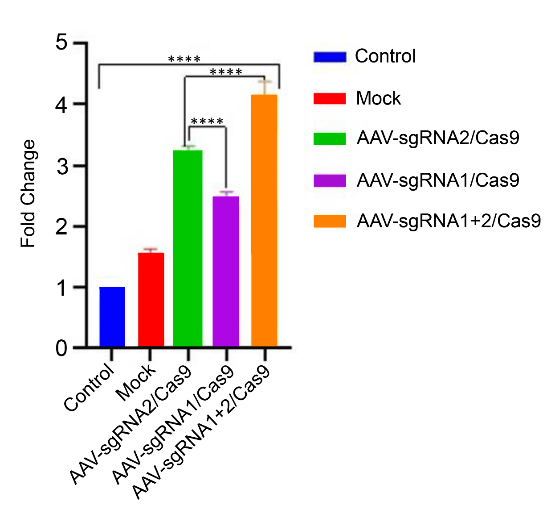


**Figure 2B.** The p53 protein level in HeLa cells was measured using western blot on day 5 after transduction. The ImageJ software was used to measure the p53:GAPDH ratios in HeLa cellsGAPDH was used as an internal control. The expression level of p53 protein was significantly increased in cells infected with AAV-E6-CRISPR/Cas9 compared with the control and mock cell groups (****p < 0.0001). The expression level of GAPDH was used to normalize the band intensity.
